# NF‐κB and HIF crosstalk in immune responses

**DOI:** 10.1111/febs.13578

**Published:** 2015-11-24

**Authors:** Laura D'Ignazio, Daniel Bandarra, Sonia Rocha

**Affiliations:** ^1^ Centre for Gene Regulation and ExpressionCollege of Life SciencesUniversity of DundeeUK

**Keywords:** B cells, HIF, hypoxia, IKK, immune response, inflammation, macrophages, neutrophils, NF‐κB, T cells

## Abstract

Hypoxia and inflammation have been associated with a number of pathological conditions, in particular inflammatory diseases. While hypoxia is mainly associated with the activation of hypoxia‐inducible factors (HIFs), inflammation activates the family of transcription factor called nuclear factor‐kappa B (NF‐κB). An extensive crosstalk between these two main molecular players involved in hypoxia and inflammation has been demonstrated. This crosstalk includes common activating stimuli, shared regulators and targets. In this review, we discuss the current understanding of the role of NF‐κB and HIF in the context of the immune response. We review the crosstalk between HIF and NF‐κB in the control of the immune response in different immune cell types including macrophages, neutrophils and B and T cells. Furthermore the importance of the molecular crosstalk between HIFs and NF‐κB for a variety of medical conditions will be discussed.

**Abbreviations**
COPD: chronic obstructive pulmonary disease
HCC: hepatocellular carcinoma
HIF: hypoxia‐inducible factor
IKK: IκB kinase
IL: interleukin
LPS: lipopolysaccharide
MMP: matrix metalloprotease
mTOR: mechanistic target of rapamycin
NF‐κB: nuclear factor‐kappa B
NO: nitric oxide
PHD: prolyl hydroxylase domain
RA: rheumatoid arthritis
STAT3: signal transducer and activator of transcription 3
TAK1: transforming growth factor β‐activated kinase 1
TAM: tumour‐associated macrophage
TCR: T cell receptor
TLR: Toll‐like receptor
VEGF: vascular endothelial growth factor
VHL: von Hippel–Lindau

## Introduction

Nuclear factor‐kappa B (NF‐κB) is considered the main pro‐inflammatory family of transcription factors involved in several relevant medical pathologies, such as rheumatoid arthritis and cancer 1, 2, 3. It was first found by Ranjan Sen and David Baltimore in 1986 in the context of the expression of a gene encoding immunoglobulin‐κ light chain in B lymphocytes 4. Since those findings, numerous reports have demonstrated the involvement of several NF‐κB subunits and their mechanisms in the context of gene expression, signalling pathways, and/or human diseases 5. In this review, we discuss the current understanding of the role of NF‐κB in the context of the immune response, with relevance to a transcription factor involved in the response to lack of oxygen (hypoxia), named hypoxia‐inducible factor (HIF). We review the crosstalk between HIF and NF‐κB in the control of the immune response and its importance for a variety of medical conditions.

## NF‐κB pathway in the immune system

**Figure 1:**
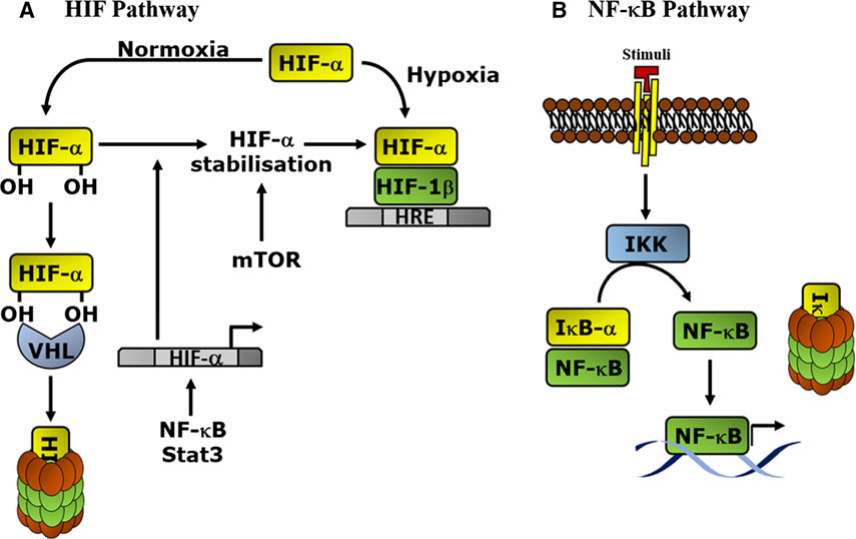
Schematic diagram of the NF‐κB and HIF activation pathways. (A) In the presence of a stimulus (such as tumour necrosis factor α, not shown), the IKK complex is activated, and mediates the phosphorylation of IκB protein, which labels it for proteasomal degradation. This action results in the release of the NF‐κB subunits (shown as NF‐κB), and translocation into the nucleus in order to activate target genes. (B) In the presence of oxygen (normoxia) HIF‐α is hydroxylated by PHD proteins (not shown), and targeted for proteasome‐mediated degradation after being polyubiquitinated by von Hippel–Lindau (VHL) protein. In the presence of low oxygen (hypoxia), HIF‐α is stabilised and interacts with the HIF‐1β subunit to activate target genes containing hypoxia response elements (HRE). In normoxia, HIF can also be stabilised by increase in its translation by mTOR, as well as through increase of its transcription by STAT3, or NF‐κB.

NF‐κB is a collective name for a family of transcription factors composed of RelA (p65), RelB, c‐Rel, NF‐κB1 (p50/p105), and NF‐κB2 (p52/p100). These subunits can be activated by many different stimuli, including bacterial lipopolysaccharide (LPS), viral pathogens, cytokines or growth factors 6. This activation involves the degradation of a family of inhibitory proteins know as IκBs. After phosphorylation of IκBs by kinases (such as the IκB kinase (IKK) complex), which respond to diverse stimuli, IκBs are targeted for degradation by the proteasome, and the NF‐κB subunits are then liberated from the inhibitory complex (Fig. 1A). NF‐κB subunits are then able to translocate to the nucleus to activate myriad NF‐κB‐specific target genes 7, 8. NF‐κB family members are capable of forming several combinations of homo‐ or heterodimers, in order to activate a complex regulatory network that culminates with the activation or repression of hundreds of genes 1, 8, 9, 10. Many of these genes, such as those for cytokines, chemokines or membrane receptors, are involved in the immune response 11, 12, 13. Generally, mammalian immune responses can be grouped into innate or adaptive 14, and in both cases the immune response starts with the recognition by the host of the presence of unfamiliar pathogens. This recognition triggers a chain of events that promotes the clearance of the pathogens. Importantly, NF‐κB acts as the commander of this immunologic response 11, although many other transcription factors are also involved. It is through the coordination of the several NF‐κB subunits and pathways that a controlled and balanced response is achieved, when cells are stimulated by an external signal 13. When NF‐κB is impaired or deregulated, this response is deficient or out of control, leading to a lack of immune response, or an excessive and damaging inflammatory response, respectively 5, 15.

NF‐κB is also important for the modulation of the proliferative balance of the immune cells. Many NF‐κB target genes, including those for Bcl‐xL, inhibitors of apoptosis protein, or granulocyte–macrophage colony‐stimulating factor, have been shown to regulate the proliferative outcome of immune cells 13. For example, NF‐κB is a key player in the control of the proliferation or cell death of hematopoietic cells. Several studies have shown the importance of NF‐κB in regulating processes involved in the rapid expansion of hematopoietic cells, such as T cells, B cells, natural killer cells and dendritic cells 16, 17, 18, 19, 20.

## HIF pathway in the immune system

The HIF family is a master regulator of the cellular response to low oxygen or hypoxia 21. HIF is a heterodimeric transcription factor, encompassing a HIF‐α and a HIF‐1β subunit. Three different HIF‐α subunits have been found (HIF‐1α, HIF‐2α, HIF‐3α), which have some overlapping but mostly distinct functions in the cell 22. Oxygen sensitivity is accomplished through the action of a class of dioxygenase that modify the HIF‐α subunits specifically. As such, prolyl hydroxylase domain (PHD) proteins (PHD1, PHD2, and PHD3) hydroxylate key proline residues within the oxygen‐dependent degradation domain of the HIF‐α subunits, creating a high‐affinity binding site for the E3‐ligase complex containing the tumour suppressor von Hippel–Lindau (VHL). This results in ubiquitination and proteasome‐dependent degradation of HIF‐α, when oxygen is present 23. In addition, an asparagine hydroxylase (factor inhibiting HIF; FIH), hydroxylates a key asparagine within the transactivation domain of HIF‐α, preventing the recruitment of coactivators CREB binding protein (CBP) and/or p300 24. The lack of association between HIF and CBP/p300 results in reduced transcriptional activity of the complex under normoxia or moderate hypoxia 24. In response to hypoxia, HIF transactivates a variety of genes involved in many processes such as glycolysis, angiogenesis, proliferation, migration, autophagy and apoptosis, amongst others 21.

Although HIF is mainly associated with the hypoxia response, the last few years have demonstrated a role for HIF in the inflammation response. Numerous studies have shown that HIF‐1α is induced in response to infection by different pathogens and in several immune cell types 22, 25. Similarly, recent studies have alluded to the fact that HIF‐1β and HIF‐2α are also involved in the control of the immune responses in cells, and in model organisms, such as zebrafish and mouse 26, 27. HIF is also able to induce a number of pro‐inflammatory cytokines and chemokines directly, further contributing to the inflammation response.

However, while it is possible that HIF induction follows a similar pathway to that of the hypoxia response, additional mechanisms for HIF induction, independent of oxygen level, have also been demonstrated (Fig. 1B). These include, increased translation via a mechanistic target of rapamycin (mTOR)‐dependent mechanism 28, but also increased transcription in a manner dependent on either signal transducer and activator of transcription 3 (STAT3) 29 or more commonly NF‐κB 22. Importantly, HIF‐mediated control of the immune responses involves an intricate crosstalk with NF‐κB.

## HIF and NF‐κB crosstalk

Hypoxia and inflammation have been associated with a number of pathological conditions, in particular inflammatory diseases 30. As part of this complex interaction, an extensive crosstalk between the two main molecular players involved, HIF (hypoxia) and NF‐κB (inflammation), has been reported. This crosstalk includes common activating stimuli, and shared regulators and targets, as recently reviewed 31. The hypoxia‐induced activation of the NF‐κB pathway was first reported in 1994 32, and thereafter a number of studies followed to elucidate the mechanisms underlying this activation. For instance, hypoxia has been identified as a common activator of both HIF and NF‐κB in an IKK–transforming growth factor β‐activated kinase 1 (TAK1)‐dependent manner 33. Also, the existence of an evolutionarily conserved negative feedback mechanism, through which HIF can regulate NF‐κB in an IKK–TAK1‐ and cell division protein kinase 6 (CDK6) ‐dependent manner, has emerged 34. On the other hand, NF‐κB has been shown to be a direct modulator of HIF expression in inflammation and hypoxia 35, 36. Despite the relatively well‐established ability of NF‐κB to induce HIF, the knowledge about HIF contributions to the NF‐κB pathway is still mainly limited to HIF‐1α. In fact, HIF‐1α can restrict NF‐κB transcriptional activity *in vivo* and *in vitro* under inflammatory conditions 34, 37, 38. Instead, to date, only scarce attention has been given to HIF‐1β and HIF‐2α subunits in the context of inflammation. However, both these HIF subunits have been associated with NF‐κB 36, 39, 40.

## HIF and NF‐κB crosstalk in the immune system

**Figure 2:**
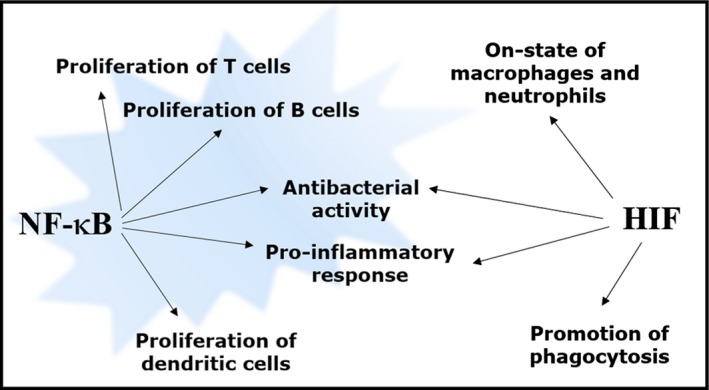
Schematic diagram of NF‐κB and HIF roles in the immune response. NF‐κB is involved in the increase of proliferation of several immune cells (T, B and dendritic cells). Additionally, NF‐κB and HIF share some functions in the activation of the immune response to infection, by promoting the expression of pro‐inflammatory cytokines, and increasing the antibacterial activity. HIF is also important for the activation of macrophages and neutrophils (on state), as well as for the promotion of phagocytosis.

Hypoxic response and innate and adaptive immunity are tightly connected. The synergistic interaction between these stress responses is mainly mediated by a regulatory loop involving the transcription factors HIF and NF‐κB (Fig. 2). For instance, macrophages infected with bacteria and mice subjected to hypoxia showed a defective HIF‐1α expression upon deletion of the IKKβ‐encoding gene 2. Furthermore, hypoxia can stimulate NF‐κB activation by negatively modulate IKKβ catalytic activity through inhibition of prolyl hydroxylases 41. Several studies suggested an important role of HIF‐1α in promoting the expression of NF‐κB‐regulated inflammatory cytokines in macrophages after LPS stimulation 42, and in mediating NF‐κB activation in anoxic neutrophils 43. Generally, while circulating in oxygen‐rich blood, myeloid‐derived phagocytes, such as neutrophils and macrophages, maintain their bactericidal and pro‐inflammatory capacities in the ‘off‐state’. Then, entering the infected tissue, in the migration across the endothelium, they encounter a decreasing oxygen gradient. In addition, the hypoxic environment characteristic of the site of infection, resulting from an elevated oxygen consumption by immune cells 44, 45, triggers an increased stabilisation of HIF‐1α protein by reduction of prolyl hydroxylase activity. Following HIF activation, the expression of innate immune response genes, containing hypoxia‐responsive elements in their promoters, increases, reaching the maximal induction through NF‐κB activation after direct pathogen encounter. This further boosts HIF‐1α transcription and enhances the host defence response. In fact, HIF activity is involved in a series of events promoting the ‘on‐state’ of the immune response, i.e. releasing pro‐inflammatory cytokines and antimicrobial peptides, promoting phagocytosis, increasing phagocyte lifespan by inhibiting apoptosis, and activating production of nitric oxide (NO; which, in turn, interferes with HIF degradation and creates an amplification loop for phagocyte activation 46). It has been reported that VHL protein‐lacking macrophages kill bacteria more efficiently than wild‐type macrophages thanks to constitutive stabilisation of HIF 47. Therefore, the concept that HIF regulates intrinsic immunity and inflammatory response underlies the development of novel pharmacological approaches. For instance, the restriction of prolyl hydroxylase access to iron, by hypoxia‐mimetic HIF‐agonists, enhances murine macrophage activity *in vitro* 47.

Considering the evidence that changes in oxygen levels regulate proliferation and differentiation of multiple cell types involved in the immune system, this review will examine the current knowledge about the intricate crosstalk between HIF and NF‐κB in neutrophils, macrophages, and T and B cells, culminating in a detailed analysis of the two stress–response systems in various disease contexts.

## HIF and NF‐κB crosstalk in macrophages

Macrophages are versatile hematopoietic cells that derive from monocytes that circulate in the bloodstream for 1–2 days before migrating into tissues and completing their differentiation. These myeloid cells are largely involved in the immune response, playing a crucial role both in innate and adaptive immunity. For instance, they initiate the inflammatory response, phagocytose and kill bacteria, and recruit lymphocytes 48. Showing extensive plasticity, macrophages can undergo two different types of activation: the classical M1 profile, induced by interferon γ or Toll‐like receptor (TLR) ligands, triggering a characteristic pro‐inflammatory activity; and the alternative M2 profile, induced by specific interleukins (i.e. interleukin (IL)‐4 and IL‐13), typical of anti‐inflammatory activity 49. During the host response, an early event is an increased monocyte extravasation, infiltration and accumulation in the inflammation site. Together with vascular damage and oedema, the intensive metabolic activity of infiltrating cells contributes to lowering of the oxygen tension in the inflamed tissue. Hypoxia represents an important factor affecting the human monocytopoiesis 50, in particular the maturation to macrophages. In fact, in response to decreased oxygen levels, monocytes switch to a glycolytic metabolism and arrest their migration induced by the chemotactic gradient. Thus, differentiated macrophages serve as phagocytes and accumulate in hypoxic inflammation sites 50. Gene expression changes, present in macrophages in hypoxic conditions *in vitro*, have been reviewed elsewhere 51. An example is the up‐regulation of molecules necessary for macrophage survival, such as glucose transporter 1 (GLUT1) 52. Both HIF‐1α and HIF‐2α importantly regulate macrophage functions in hypoxia, although it is still controversial whether they have clearly distinct roles 50. The use of myeloid specific *Hif‐1*α ablation in mouse models showed that HIF‐1α mediates macrophage‐dependent inflammatory and antibacterial activities 47, 53. A recent study demonstrated that HIF‐2α shares some functions with HIF‐1α, such as the regulation of acute inflammatory response. However, this regulation does not involve NO production or co‐stimulatory molecule expression, but promotes specific pro‐inflammatory cytokine expression, in response to M1 stimuli, and leukocyte infiltration. Of note, in human macrophages, HIF‐2α overexpression enhanced the transcription of proangiogenic genes (i.e. those for vascular endothelial growth factor (VEGF), IL‐8 and platelet‐derived growth factor β) even in normoxic conditions 48.

Interestingly, in human and murine macrophages, hypoxia can activate gene expression also in a HIF‐independent manner, through the upregulation of NF‐κB, activating transcription factor 4 (ATF4), and early growth response‐1 (Egr‐1) 51. For instance, upon hypoxia incubation, NF‐κB1 (p50) has been detected in the nuclei of monocytes, while HIF‐1α remained cytoplasmic 54. Also, as previously reported (see above, HIF and NF‐κB crosstalk in the immune system), HIF and NF‐κB synergistically respond against pathogens: in fact, macrophages, infected by Gram‐negative and Gram‐positive bacteria, are characterised by a defective HIF‐1α expression following ablation of IKKβ, essential regulator of NF‐κB activity 2.

## HIF and NF‐κB crosstalk in neutrophils

Neutrophils are important myeloid cells that phagocytose foreign pathogens and promote inflammation 55. Neutrophils are short‐lived and very motile cells, using mainly glycolysis as their source of energy 55. These cells are attracted by cytokines to sites of infection, and mediate the clearing of pathogen by phagocytosis amongst other methods 55. Several studies have reported on the NF‐κB and HIF crosstalk. Seminal work by Walmsley and coworkers has demonstrated that in neutrophils, HIF‐1α activates NF‐κB in hypoxia to increase the survival of this type of cell 43. This was further confirmed using PHD3 deletion specifically in neutrophils, leading to increased induction of HIF‐1α, resulting in enhanced survival of these cells 56. Furthermore, HIF‐1α was shown to be required for glycolysis in neutrophils 53. Although, several studies have not detected HIF‐2α levels in neutrophils, a more recent study has shown that hypoxia and bacterial infection leads to increased HIF‐2α expression in both mouse and human neutrophils 27. While it is clear that HIF‐1α is leading to activation of NF‐κB, whether NF‐κB induces HIF‐1α, HIF‐2α and HIF‐1β in these cells has not been formally demonstrated. Finally, although not directly tested it is possible that HIF‐1β might be required for both HIF‐1α and HIF‐2α actions in these cells. However, more research is needed in this context.

## HIF and NF‐κB crosstalk in T cells

Host defence to pathogen infections depends on coordinated responses of various immune cell types. Particularly, T cells are lymphocytes acting as regulators and effectors of cell‐mediated immune responses. They mature as naïve conventional T cells in the thymus, and, migrating to the periphery, differentiate into several effector subsets upon encountering certain antigens or environmental conditions 57. When T cells are recruited from secondary lymphoid to sites of inflammation, they are exposed to dramatic changes in tissue signals: cytokines, chemokines, balance of nutrients and oxygen available in the new microenvironment can massively affect T cell metabolism and function 58. During development and maturation, T cells encounter different levels of oxygen tension 59. For instance, at the site of inflammation or in non‐lymphoid tissues, effector T cells may be exposed to prolonged hypoxic environments, resulting in HIF‐1α protein stabilisation. Interestingly, in T lymphocytes HIF‐1α protein expression can be robustly induced also by T cell receptor (TCR) stimulation, followed by activation of PI3 kinase/mTOR pathway 28. Alternatively, the pro‐inflammatory cytokine IL‐6 drives HIF‐1α expression by activation of STAT3 transcription factor 29. Hypoxia can induce chemokines to selectively recruit certain T cell subsets, and alter T cell stimulation capacity by changing their antigen presentation 46. A number of studies assessed the role of HIF‐1α in VHL protein‐deficient thymocytes 60 or HIF‐1α‐deficient T cells. Particularly, in this context, an increased inflammatory cell infiltration and vascular remodelling were observed 61, suggesting HIF‐1α as a T cell negative regulator.

Interestingly, stimuli inducing HIF‐1α (i.e. cytokines and antigen stimulation) also activate NF‐κB. In murine, HIF‐1α‐deficient T cells, an enhanced NF‐κB activity (both in p50 and RelA) was observed upon cecal ligation 62. The engagement of TCR, and coreceptor CD28, can also activate the NF‐κB pathway 63, inducing, in turn, the transcriptional activation of a plethora of targets, such as IL‐2, a cytokine involved in T cell proliferation and survival 57. Therefore, NF‐κB is one of the most important transcription factors contributing to T cells’ development and differentiation. The majority of the studies designed to uncover the role of NF‐κB in T cell activation, expansion and regulation involved genetic models of systemic or conditional ablation of IKK proteins, NF‐κB subunits, and adapter components of the pathway. In T cell development, NF‐κB is involved already in the early antigen‐independent phase of thymocyte differentiation, as well as the final antigen‐dependent lineage commitment and postselection maturation 20. Further evidence of the crosstalk between HIF and NF‐κB is that both pathways are essential in differentiation of regulatory T cells (Tregs), by direct transcriptional activation of Foxp3 64, 65, in which cRel and RelA/p65 are particularly involved 20, 66. In addition, HIF‐1α also promotes the fine balance between Tregs and the pro‐inflammatory Th17 subset 29, 67. In fact, during Th17 differentiation CD4^+^ T cells rely on HIF‐1α to regulate cell metabolism 67.

Moreover, a recent study reported that T cells are more sensitive to hypoxia upon stimulation of NF‐κB, showing an enhanced HIF‐1α‐dependent adenosinergic signalling 68. Therefore, taking into account the numerous shared activators and targets between HIF and NF‐κB, a better understanding of their crosstalk in T cell development and function is necessary to specifically target the pathways in novel therapeutic strategies.

## HIF and NF‐κB crosstalk in B cells

While it is clear that NF‐κB is required for normal B cell development and it is often found deregulated in B cell malignancies 69, only recent work has demonstrated the importance of HIF in B cells. Genetic studies using lineage‐specific deletion of HIF‐1α in mice have shown that HIF‐1α is required for normal B cell development 70, 71. In fact, HIF‐1α deletion resulted in autoimmunity in chimeric mouse models 70. However, whether HIF and NF‐κB crosstalk in the cell system has only been determined in the context of disease. In particular, NF‐κB was shown to be responsible for the induction of HIF‐1α mRNA in malignant lymphoma 72. No information is available regarding how HIF modulates NF‐κB function under these cells. In addition, other HIF isoforms have not been investigated as well. It is possible, however, that HIF‐2α might be involved in B‐cell lymphomas, as a recent study reported that HIF‐2α mRNA is controlled by E2F1 in cells, and elevated in a mouse model of B‐cell lymphoma 73. However, much more work is needed to fully investigate the HIF–NF‐κB crosstalk in this cell type.

## HIF and NF‐κB crosstalk in disease conditions

As mentioned above there is an intimate interaction between HIF and NF‐κB, in immune cells. Importantly there are various examples of this crosstalk in the immune response occurring in disease conditions. These include rheumatoid arthritis (RA) 74, asthma and chronic obstructive pulmonary disease (COPD) 75, and cancer 76.

### Crosstalk in rheumatoid arthritis

RA is a progressive autoimmune disorder caused by a chronic inflammation of the synovium. Its characteristic clinical features are pain, stiffness, swelling and joint destruction. Normally the innate immune system recognises microbial pathogens by action of macrophages and dendritic cells, triggering the production of specific inflammatory cytokines and chemokines. Then, innate immune system cells can travel to local lymphoid tissues to initiate the adaptive response when necessary. In RA the innate immune system is pathologically continuously activated. In fact, the persistent interaction among synoviocytes and innate (i.e. macrophages, dendritic cells, etc.) or adaptive immune cells (B and T lymphocytes) has been reported 74. As previously described (see above, HIF and NF‐κB crosstalk in macrophages), macrophages are more numerous at the inflamed site, because of an elevated chemotaxis and reduced apoptosis 77. Consequently, macrophages are highly abundant in RA synovium. In the RA joints, macrophages can promote inflammatory processes, such as lymphocyte invasion, angiogenesis and secretion of matrix metalloproteases (MMPs). In addition, the expanded metabolic activity characteristic of the RA synovium contributes to unbalancing oxygen homeostasis, enhancing hypoxia in the microenvironment. On the other hand, hypoxia itself is able to intensify macrophage pro‐inflammatory capacity by a positive regulatory loop. Confirming the pathological relevance of HIF‐1α expression by macrophages in RA synovium, HIF‐1α conditional knockout mice showed lower infiltration of myeloid cells 53. RA synovium is also enriched in protein of complement system, and autocrine and paracrine‐acting pro‐inflammatory cytokines, mainly expressed by macrophages. Among them, TNF‐α, IL‐1β and IL‐6, which play an important role in the progression of the disease and are transcriptionally upregulated by hypoxia as well. Interestingly, TNF‐α and IL‐1β expression is further induced by IL‐17, produced by T helper cells. IL‐17 contributes to the inflammatory response by induction of neutrophil recruitment and NF‐κB activation 78. All this evidence supports the existence of an extensive crosstalk between HIF and NF‐κB in RA, as recently reviewed 30. A better understanding of the molecular interaction between the two transcription factors will be particularly relevant for developing novel therapeutic strategies. The majority of therapies currently in use are based on TNF‐α inhibitors. A treatment with recombinant human IL‐11 was shown to decrease not only the production of TNF‐α, but also NF‐κB activity 79. The same decreased TNF‐α production has been observed in macrophages upon treatment with PHD inhibitors, in an NF‐κB‐dependent but HIF‐1α‐independent manner 50. A novel promising therapeutic approach might promote the selective activation of an M2 macrophage (anti‐inflammatory) profile, considering that targeting specific macrophages would avoid side‐effects connected to a systemic depletion, as well as allowing the reaching of remote inflammation sites.

### Crosstalk in asthma and chronic obstructive pulmonary disease

Asthma is a chronic inflammatory disorder of airways, in which key symptoms are wheezing, coughing, shortness of breath, and sputum production. Chronic obstructive pulmonary disease (COPD) is an obstructive lung disease characterised by narrowing of bronchioles, limited airflow, and emphysema (irreversible of damage to lung tissue) 75. Supporting the fact that inflammation is a main feature of both diseases, the vast majority of patients are currently treated by anti‐inflammatory glucocorticoids, which are, unfortunately, ineffective in the most severe cases. Glucocorticoids target specifically the transcription factor NF‐κB 80 that, in asthma and COPD, is activated by TNF‐α, IL‐1β or TLRs. In turn, NF‐κB controls the infiltration of inflammatory cells in airways tissues, by regulating the expression of cytokines, chemokines and cell adhesion molecules 75. Asthmatic tissues are also characterised by mucous cell metaplasia, as well as inflammation mediated by eosinophilic and T helper 2 (Th2) cells 81. Alternatively, airway inflammation can involve a higher number of neutrophils, and, thus, more Th1 lymphocytes. In general, asthmatics and individuals affected by COPD showed higher NF‐κB DNA binding, following increased degradation of the IκB inhibitor 82, 83. In fact, NF‐κB has a pivotal role in the regulation of inflammatory gene expression in airway cells. For instance, it regulates IL‐2 and IL‐4 production, respectively in Th1 and Th2 cells. Also, it controls IL‐8 expression both in neutrophils and macrophages 75. A deep understanding of the NF‐κB‐mediated regulatory mechanism associated with neutrophilic response in asthma and COPD patients is relevant to overcoming the related steroid resistance. To date, several efforts have been made in the targeting of NF‐κB pathway intermediates in an experimental mouse model of asthma and COPD 75. However, this field of research needs further investigations, especially considering a possible crosstalk with hypoxia. The role of HIF in asthma is still controversial, and in most cases associated specifically with allergic asthma. Upregulation of HIF‐1α and HIF‐2α occurs in mouse model of allergic airway inflammation together with an increase in eosinophil recruitment and VEGF expression 84. These few preliminary results open new possibilities of asthma treatment by using inhibitors of HIF; however, additional research is needed.

### Crosstalk in cancer

Recent findings firmly establish that HIF is involved in tumourigenesis, interacting with a variety of other transcription factors, such as STAT3, Myc, Notch, and NF‐κB 76. Particularly, increased expression of HIF‐1α has been associated with metastasis and poor prognosis in a number of cancer subtypes. Neoplastic areas show a high level of hypoxia, producing, in turn, pro‐inflammatory mediators responsible for the recruitment of more immune cells at tumour sites. Frequently, this results in chronic inflammation, with a relevant activation of NF‐κB. Several reports support a collaboration between HIF and NF‐κB in tumourigenesis promotion, through activation of genes (i.e. genes for IL‐6, cyclooxygenase 2, MMP‐9, etc.), as well as in induction of prosurvival genes, such as that for Bcl‐2 76. In addition, HIF‐1α controls the tumour‐associated inflammatory response mediating activation of TLR4. A classic example of cancer characterised by chronic inflammation is the hepatocellular carcinoma (HCC) 85. A key feature of this cancer type is the constant expression of cytokines recruiting immune cells to the liver. In this context, an important role is played by TNF‐α, activated directly by NF‐κB, and by tumour‐associated macrophages (TAMs). They are the main component of leukocyte infiltration, and, due to their plasticity, TAMs own both pro‐ and anti‐inflammatory properties. For example, inhibition of TNF‐α resulted in a reduction of HCC cells, attributing a protumoural function to NF‐κB activation in macrophages 86. According to this, TAMs isolated from NF‐κB1^–/–^ mice showed a preferred activation of antitumoural M1 expression profile 87. However, evidence about the role of TAMs is still controversial, and further clarification is needed, taking into consideration that TAMs apparently accumulate in scarcely vascularised and hypoxic regions. Expression of HIF‐1α and HIF‐2α in TAMs seems to be involved in HCC progression with overlapping functions 48, 88. Therefore, macrophages are currently considered a possible effective vehicle to target hypoxia‐regulated genes in solid tumours, otherwise not accessible to classic therapeutic agents circulating in the bloodstream.

### Potential for new therapeutic approaches targeting the HIF‐NF‐κB crosstalk

Given the crosstalk described above, it is plausible to think that targeting the HIF pathway will impact on NF‐κB function. In fact, a number of studies using both genetic and chemical inhibition of the PHD proteins have demonstrated therapeutic effects in disease models such as colitis and even in response to infection 89, 90, where inflammation and NF‐κB play an important role. It would therefore be interesting to expand the possible therapeutic effect of both HIF activators and inhibitors to other diseases of the immune system such as RA and COPD. However, targeting of the individual HIF‐α isoforms might be required to achieve the desired therapeutic effects. Additional work, using the available HIF modulators in these disease models, is necessary to test the efficacy of these drugs in different settings, and such work could be very informative in the understanding of what form HIF–NF‐κB crosstalk takes in diverse cell types.

## Summary

**Figure 3:**
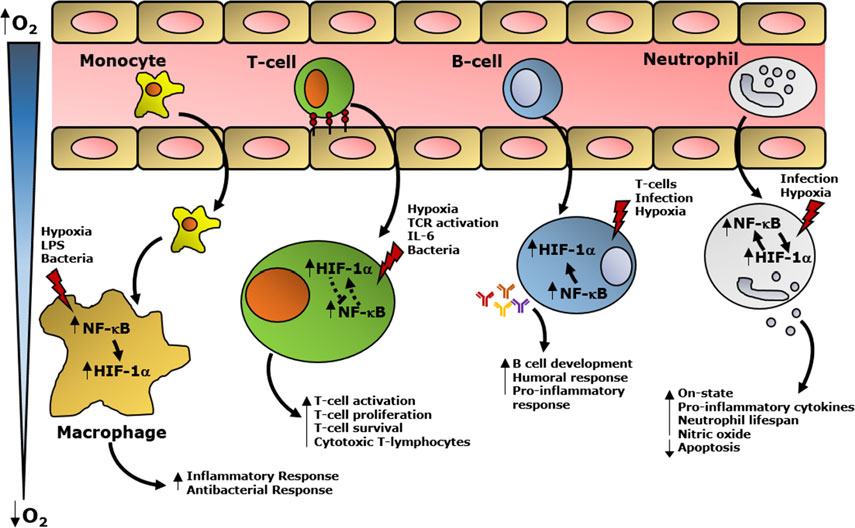
HIF and NF‐κB crosstalk in several immune cells. In macrophages NF‐κB increases the transcription of HIF‐1α in response to hypoxia, LPS, or bacterial infection. The increase of both NF‐κB and HIF leads to the increase of an inflammatory response by enhancing the expression of pro‐inflammatory cytokines. In T cells, HIF was shown to have a role in restraining the NF‐κB activity. Upon hypoxia, TCR activation, IL‐6, or bacterial infections, HIF and NF‐κB are increased, leading to the enhancement of T cell proliferation, survival and activation. Hypoxia and stimulation by infection can also increase HIF activity through the activation of NF‐κB, resulting in an increase in the development of B cells, and the humoral response. In neutrophils HIF enhances NF‐κB activity. The increase of these transcription factors promote the on‐state of neutrophils, leading to an increase of nitric oxide and pro‐inflammatory cytokine production, as well as the reduction of cellular apoptosis.

An intricate and complex crosstalk between HIF and NF‐κB exist in many cell types (Fig. 3). The ultimate outcome is very much cell type dependent. While in certain cell types, these transcription factors cooperate, which is evident in the case of neutrophils, in other cells, they antagonise each other such as in subtypes of T cells (Fig. 3). This, however, presents an opportunity for specialised clinical intervention, as several HIF agonists that could be used for immune‐related diseases are in clinical development.

## Author contributions

L.D'I., D.B. and S.R. all wrote the manuscript. S.R. compiled the final edits.
